# Awareness of HIV Testing Guidelines Is Low among Swiss Emergency Doctors: A Survey of Five Teaching Hospitals in French-Speaking Switzerland

**DOI:** 10.1371/journal.pone.0072812

**Published:** 2013-09-06

**Authors:** Katharine E. A. Darling, Nathalie de Allegri, Daniel Fishman, Reza Kehtari, Olivier T. Rutschmann, Matthias Cavassini, Olivier Hugli

**Affiliations:** 1 Infectious Diseases Service, Lausanne University Hospital, Lausanne, Switzerland; 2 University of Lausanne, Lausanne, Switzerland; 3 Hôpital Fribourgeois Régional, Fribourg, Switzerland; 4 Hôpital de Sion, Sion, Switzerland; 5 Hôpitaux Neuchâtelois, sites de Pourtalès et de la Chaux-de-Fonds, Neuchâtel, Switzerland; 6 Hôpitaux Universitaires de Genève, Geneva, Switzerland; 7 Emergency Department, Lausanne University Hospital, Lausanne, Switzerland; University of Pittsburgh Medical Center, United States of America

## Abstract

**Background:**

In Switzerland, 30% of HIV-infected individuals are diagnosed late. To optimize HIV testing, the Swiss Federal Office of Public Health (FOPH) updated ‘Provider Induced Counseling and Testing’ (PICT) recommendations in 2010. These permit doctors to test patients if HIV infection is suspected, without explicit consent or pre-test counseling; patients should nonetheless be informed that testing will be performed. We examined awareness of these updated recommendations among emergency department (ED) doctors.

**Methods:**

We conducted a questionnaire-based survey among 167 ED doctors at five teaching hospitals in French-Speaking Switzerland between 1^st^ May and 31^st^ July 2011. For 25 clinical scenarios, participants had to state whether HIV testing was indicated or whether patient consent or pre-test counseling was required. We asked how many HIV tests participants had requested in the previous month, and whether they were aware of the FOPH testing recommendations.

**Results:**

144/167 doctors (88%) returned the questionnaire. Median postgraduate experience was 6.5 years (interquartile range [IQR] 3; 12). Mean percentage of correct answers was 59 ± 11%, senior doctors scoring higher (*P*=0.001). Lowest-scoring questions pertained to acute HIV infection and scenarios where patient consent was not required. Median number of test requests was 1 (IQR 0-2, range 0-10). Only 26/144 (18%) of participants were aware of the updated FOPH recommendations. Those aware had higher scores (*P*=0.001) but did not perform more HIV tests.

**Conclusions:**

Swiss ED doctors are not aware of the national HIV testing recommendations and rarely perform HIV tests. Improved recommendation dissemination and adherence is required if ED doctors are to contribute to earlier HIV diagnoses.

## Introduction

It is now well-recognized that late diagnosis of HIV in infected individuals is associated with an avoidable burden of morbidity [1], mortality and cost [2]. In spite of this, in Switzerland, 30% of individuals newly diagnosed with HIV are diagnosed late, that is, with CD4 counts <200 cells/microlitre [1]. HIV prevalence in Switzerland is 0.4% [3], above the threshold of 0.1% set by the 2006 Centers of Disease Control and Prevention (CDC) recommendations for proposing opt-out testing [4].

In March 2010, the Swiss Federal Office of Public Health (FOPH) updated the 2007 HIV testing recommendations proposing ‘Provider Induced Counseling and Testing’ (PICT) [5] (also known as ‘Physician-Initiated Counseling and Testing’). These recommendations implicitly propose diagnostic testing and targeted screening rather than opt-out testing but, like the CDC recommendations, they decouple testing from pre-test counseling and consent, whilst maintaining a need to inform the patient when HIV testing is indicated. They place the duty of testing on the doctor and emphasize the importance of recognizing four scenarios: 1) symptoms and signs of acute HIV infection (AHI); 2) those of advanced HIV infection; 3) settings in which HIV screening is indicated; and 4) patients at high risk of HIV infection. These scenarios are detailed further both in the text and in the form of five sets of lists (Table S1, S2a, S2b, S2c and S3). For patients presenting symptoms and signs of HIV infection, or in settings in which HIV screening is indicated, the revised recommendations state that ‘the doctor should inform the patient that an HIV test is indicated’ and that the patient has to state explicitly that s/he refuses to be tested for the test not to be performed. For patients considered at risk of HIV infection, through high risk activities or their socio-demographic profiles, the doctor should perform pre-test counseling and the patient must give explicit verbal consent for the test to be performed. Although the symptoms, signs and pathologies in which HIV testing is indicated are clearly listed, the difference between informing a patient that an HIV test is indicated and suggesting an HIV test after pre-test counseling might be considered as subtle.

We recently reported that the new FOPH recommendations made no difference to testing practices in nine clinical services in our centre and observed that, of the tens of thousands of patients seen in our emergency department (ED) annually, only 1% are tested for HIV [6]. In the present study, as a first step in determining why ED testing rates are so low, we set out to examine whether ED doctors are aware of the new recommendations.

## Methods

### Ethics statement

The study was approved by the ethics committee on human scientific research of the canton of Vaud, Switzerland (14^th^ February 2011). All participants (see below) provided informed written consent prior to taking part in the study.

### Setting and participants

The study took place in the EDs of the five principal hospitals in French-speaking Switzerland: two university hospitals (Lausanne University Hospital and the Hôpitaux Universitaires de Genève) and three cantonal (university-affiliated) hospitals (Hôpitaux Neuchâtelois, the Hôpital de Sion, and the Hôpital Fribourgeois). Between them, the EDs in these hospitals receive over 175,000 patient visits per year (Table S4). All ED doctors of Resident, Chief Resident and Attending grades were invited to participate in the study. No specific training on HIV testing was organized for medical staff prior to the study so as to be representative of the ‘real life’ situation. However, all junior doctors have received training on this subject as part of their undergraduate studies (MC, personal communication). Because we aimed to target only healthcare professionals who are directly responsible for requesting HIV tests, medical students and non-medical ED staff, who do not have this responsibility, were excluded.

It should be noted that, in Switzerland, emergency medicine is accredited as a sub-specialty rather than a full specialty and so there is no formal national training curriculum. Training received by junior doctors in the ED is determined by the areas of expertise of their supervising colleagues and by the clinical competencies perceived to be required in the specific ED [7].

### Study design

The survey took place between 1^st^ May and 31^st^ July 2011. ED doctors were asked to complete the questionnaire (see below) during scheduled departmental seminar times organized by each ED head. The estimated time to complete the questionnaire was 20 minutes and we aimed for a minimum participation of 65%. The ED heads were also requested to provide figures for patient turnover in 2010 and the number of doctors practicing in their departments. Completed questionnaires were returned to the Lausanne centre for incorporation into an anonymized database.

### Questionnaire (Text S1 and Table 1)

As all participants were French-speaking, the study questionnaire was written and completed in French. An English translation is supplied as Text S1. The questionnaire consisted of a front page and three sections of questions. The front page covered participant demographics, including place of work, postgraduate experience (in years) and grade (options: ‘Resident’, ‘Chief Resident’ and ‘Attending’). The first section of questions (Section 1) contained 12 clinical scenarios describing symptoms and/or pathologies based on the lists provided in the FOPH recommendations. For each scenario, participants had to state a) whether or not HIV testing was indicated according to the FOPH recommendations (options: ‘yes’ and ‘no’) and b) the probability with which they would perform an HIV test in their own clinical practice (options: 0-25%, 26-50%, 51-75% and 76-100%). A *disparity* was said to occur if we observed a mismatch between testing indication awareness and clinical practice, that is, if a participant answered correctly that an HIV test was indicated but stated the probability of performing a test in practice at 0-25% or, conversely, that an HIV test was not indicated but stated the probability of performing a test in practice at 75-100%. The second section (Section 2) contained seven questions requiring the participants to identify the correct method of testing, either informing patients that HIV testing was indicated or performing pre-test counseling, and six questions requiring participants to identify the serological tests with which HIV testing should be performed routinely. As for Section 1, all questions in Section 2 were derived from the FOPH recommendations. Each question in Sections 1 and 2 was assigned one point if answered correctly and scores were presented as a percentage of the maximum possible score: 12 points for Section 1; seven points for the consent part of Section 2; six points for the serology part of Sections 2, and 25 points in total.

The final section of the questionnaire asked how many HIV tests participants had performed in the preceding four weeks and whether or not they were aware of the updated FOPH recommendations. If aware, they were invited to state the source (options: clinical seminar, on line search, FOPH recommendations, a specific article on HIV testing published in a national journal: the *Swiss Medical Forum*, or ‘other’ means).

Prior to this study, the questionnaire was pilot-tested on five senior doctors working in acute medicine at a clinic not included in our study. The purpose of this pilot was two-fold: 1) to ensure the questions were fully comprehensible and without ambiguity and 2) to determine the time required to complete the questionnaire. As the five doctors completed all sections correctly and reported no ambiguity, the questionnaire was used for the study without further validation.

### Statistical analyses

Data are presented as means with standard deviation (SD), medians with interquartile range (IQR), or percentages. Proportions were compared in two-way contingency table analyses using Chi squared tests and means were compared using Student’s t test. Non-parametric data were analyzed using the Mann Whitney U test. All statistical analyses were performed using STATA™ software version 12 for Windows (StataCorp, College Station, Texas) and Microsoft Excel 2008 (Microsoft Corporation, Redmond, WA, USA).

## Results

### Participant characteristics

Of 164 ED doctors invited to participate in the study, 144/164 (88%) completed the questionnaire. Response rate was >65% in all but one centre (where response rate was 43%, Table S4). Of the 144 participants, 75/144 (52%) were male, 25/144 (17%) were Attendings and 100/144 (69%) worked at university (as opposed to cantonal) hospitals. Mean age was 35 +/- 6 years and median postgraduate experience was 6.5 years (IQR 3; 12).

### Questionnaire responses (Tables 1, 2 and S4)

Questionnaire scores (the percentage of correct responses) were examined according to clinical scenario (Table 1), doctor grade (Table 2) and ED (Table S4). In Section 1, the mean score for all participants was 66 ± 14%. Participants scored better for clinical scenarios in which HIV testing was not indicated than for those in which testing was indicated (88% ± 13% versus 59% ± 15%, *P*=0.04). The lowest scoring question in this section was question 2 (correct response rate of 26%, Table 1), which pertained to AHI.

**Table 1:** Clinical scenarios in Sections 1 and 2 of the study questionnaire, whether HIV testing is indicated according to the 2010 Swiss Federal Office of Public Health recommendations, number (percentage) of correct responses for each question and number (percentage) of questions with disparity, as defined in Methods.

Question	Clinical scenario	Correct response	Correct responders, n (%)	Disparity, n (%)
Section 1				
	**Is HIV testing indicated in the following situations, according to national recommendations:**			
1	Acute peripheral facial nerve palsy	Yes	72 (50)	12/72 (17)
2	3-day history of sore throat, fever, submandibular lymphadenopathy; no cough	Yes	37 (26)	5/37 (14)
3	Fever, headache, meningeal signs; Gram negative diplococci on CSF examination	No	98 (68)	0/98 (0)
4	3-month history of unintentional weight loss, diarrhea and intermittent fever	Yes	111 (77)	4/111 (3.6)
5	Urosepsis in elderly woman with indwelling catheter	No	140 (97)	0/140 (0)
6	Several-month history of general fatigue	Yes	48 (33)	8/48 (17)
7	Fractured wrist post-trauma	No	140 (97)	0/140 (0)
8	Aseptic meningitis in subfebrile patient	Yes	110 (76)	8/110 (7.3)
9	Male with dysuria and meatal erythema with no relevant sexual history	Yes	101 (70)	5/101 (5)
10	Acute rash and low-grade fever in patient immune to measles	Yes	92 (64)	9/92 (9.8)
11	Herpes zoster in young patient	Yes	105 (73)	9/105 (8.6)
12	Diagnosis of pregnancy in patient presenting with vomiting	Yes	87 (60)	12/87 (14)
Section 2				
	**Can physicians inform patients that HIV test will be performed or must explicit consent be sought**: (multiple choice)			
13	Married man presenting with features of seroconversion illness	Patient should be informed that test will be performed	35 (24)	
14	HIV suspected as causal diagnosis		21 (15)	
	**Is pre-test counseling indicated in the following settings:**			
15	Patient wishing to be tested for HIV	Yes	123 (85)	
16	Patient discloses he is homosexual	Yes	78 (54)	
17	Clinical features of acute HIV infection	No	37 (26)	
18	Patient with Kaposi’s sarcoma	No	49 (34)	
19	Patient from sub-Saharan Africa	Yes	57 (40)	
	Should an HIV test be added automatically when screening for the following conditions:			
20	Hepatitis B	Yes	125 (87)	
21	Hepatitis A	No	124 (86)	
22	Lyme disease for unexplained atrio-ventricular block	No	117 (81)	
23	Epstein-Barr virus	Yes	75 (52)	
24	Cytomegalovirus	Yes	83 (58)	
25	Causes of unexplained rash with fever	Yes	75 (52)	

We observed some disparity between participants’ awareness that HIV testing was indicated and their stated probability of performing a test in clinical practice. Disparity was more frequently observed among low-scoring questions. Of the three questions with a correct response rate of ≤50% (questions 1, 2 and 6, Table 1), 14-17% of the participants who did respond correctly that HIV testing was indicated stated a probability of 0-25% of performing a test in clinical practice. The majority (75-83%) of participants displaying this disparity were of senior grade (Chief Residents and Attendings as opposed to Residents, *P*=0.02). The only other question with a disparity rate of >10% but with a correct response rate >50% was scenario 12, a patient presenting with symptoms of pregnancy: of 60% of participants who answered correctly that HIV testing was indicated, 14% stated a 0-25% probability of conducting a test in clinical practice. Conversely, for questions in which doctors stated the guidelines did not recommend testing, no participant stated s/he would perform a test with a high probability (75-100%).

In Section 2, the mean score was 40 ± 16% for the consent questions and 69 ± 18% for the serology questions. Consent-based questions had the worst scores in this section, particularly those pertaining to the lack of requirement for pre-test counseling when HIV infection was suspected (Table 1).

Considering doctor grade, senior doctors (Chief Residents and Attendings) had a higher mean score than juniors (Residents) for Section 1 (70 ± 14% versus 63 ± 15%, *P*=0.013), Sections 1 and 2 together (64 ± 10% versus 56 ± 11%, *P*=0.001) and the serology part of Section 2 (74 ± 16% versus 66 ± 19%, *P*=0.03) but not significantly so for the consent part of Section 2 (43 ± 15% versus 37 ± 16%, *P*=0.07). Examining hospital type, there was no significant difference between the scores of university hospitals compared to non-university hospitals: 66 ± 14% versus 67± 15%, (*P*=0.71) for Section 1, but there was a difference for Section 2, for both parts: consent (34 ± 15% versus 42 ± 16%, *P*=0.03) and serology (62 ± 19% versus 72 ± 18%, *P*=0.009).

### Awareness of the FOPH HIV testing guidelines

Twenty-six participants out of 144 (18%) were aware of the new recommendations. Recommendation awareness was lower among junior doctors (Residents) than seniors (Chief Residents and Attendings) (Table 2, *P*=0.02). Considering hospital type, although none of the doctors in two of the three cantonal hospitals were aware of the recommendations (Table S4), there was no significant difference in doctor awareness between university and non-university hospitals (*P*=0.49). For doctors who were aware of the recommendations, the principal means of awareness were through reading an article on testing in the *Swiss Medical Forum* (10/26, 39%) and attending clinical seminars (9/26, 35%). Only 4/26 (15%, or 2.8% [4/144] of all participants) were aware through reading the original March 2010 FOPH bulletin. Doctors aware of the recommendations had a higher total questionnaire score (67 ± 11% versus 58 ± 11%, *P*=0.001) and a higher score for questionnaire Section 1 (76 ± 9.7% versus 64 ± 14%, *P*=0.001). Examining the questions in Section 2 regarding patient consent (which had the lowest mean score), doctors who were aware of the FOPH guidelines scored better than those unaware: 50 ± 18% versus 37 ± 16% (*P*=0.004); there was no significant difference in scores for questions regarding serology (71 ± 16% versus 69 ± 18%, *P*=0.6).

**Table 2:** Results presented according to emergency department doctors participating in the study: participant characteristics, questionnaire scores (percentage of correct responses), number of HIV tests requested by participants, and awareness of national HIV testing recommendations.

Characteristic	Resident (n = 83)	Chief Resident (n = 36)	Attending (n = 25)	All doctors (n = 144)
Age, years, mean (SD)	30.1 (2.4)	37.4 (3.9)	47.6 (5.8)	35 (6.3)
Postgraduate experience, years, median (IQR)	3 (2;5)	10.1 (8;13)	18.9 (14;23)	6.5 (3;12)
University hospital based, n (%)	57 (69)	27 (75)	16 (64)	100 (69)
Questionnaire score (% correct responses):				
Section 1 : scenarios, mean (SD)	63 (15)	71 (16)	69 (11)	66 (14)
Section 2 : consent, mean (SD)	37 (16)	41 (14)	47 (17)	40 (16)
Section 2 : serology, mean (SD)	66 (19)	76 (16)	70 (16)	69 (18)
Total (Sections 1+2), mean (SD)	56 (11)	64 (11)	63 (9.3)	59 (11)
Participants stating probability of 0-25% of requesting HIV test even if indicated, n (%)	17 (21)	14 (39)	8 (32)	39 (27)
No. of tests in past month, median (IQR)	0 (0;2)	1(0;2.3)	0 (0;2)	1 (0;2)
Aware of recommendations, n (%)	7 (8.4)	8 (22)	11 (44)	26 (18)

Abbreviations: IQR, interquartile range; SD, standard deviation

### HIV testing practices

Despite higher questionnaire scores among doctors aware of the testing recommendations, being aware did not influence the number of tests performed during the preceding four weeks (mean 1.5 versus 1.4, *P*=0.55 or median 1 [IQR 0; 2, range 1-10] in both groups, *P*=0.54). Although senior doctors tested slightly more than junior grades, this difference was not significant (*P*=0.2). There was no difference in test request numbers between university and non-university hospitals (*P*=0.8).

## Discussion

We observe that awareness of the updated national HIV testing recommendations among ED doctors in the five principal hospitals in French-speaking Switzerland is low (18%). Doctors aware of the recommendations had higher questionnaire scores for recognizing when HIV testing was indicated (Section 1) and whether or not consent was required (Section 2) but did not perform more tests than those unaware. Finally, we observe important disparities between recommendation awareness and application.

Low awareness of HIV testing recommendations among health professionals has been described. In a questionnaire-based study conducted in San Francisco, Cohan et al. observed that 44/224 non-primary care providers (24%), from departments including internal medicine, surgery, obstetrics/gynecology and emergency medicine, were aware of the 2006 CDC HIV testing recommendations [8]. Whilst it is reassuring to observe in our study that doctors who were aware of national recommendations were better at identifying the clinical settings in which HIV testing is indicated, the lack of awareness in our EDs is a problem.

Between them, the EDs we studied receive over 175,000 patient visits annually. A proportion of these patients use the ED as a source of primary healthcare, particularly individuals from vulnerable populations who have suboptimal access to healthcare and yet may be at high risk of HIV infection [9]. If we assume that all patients attending the EDs we studied are representative of the wider Swiss population, in which HIV seroprevalence is 0.4% [3], then, among the patients presenting to these centers, we would expect 525-700 to be HIV positive (based on Lausanne ED figures, up to 25% of patients present more than once per annum [OH, personal communication]). Using our figures for patient visits per annum (Table S4), we can estimate the HIV testing rate in the EDs we studied (=[tests performed / patients seen] x 100 [6]) by extrapolating the median number of tests performed per doctor per month (=1 test) to the number performed in one year (=1 x 12), and then multiplying by the number of practicing ED doctors. Based on our data in Table S4, the estimated testing rate is 1.1-1.5%. As many HIV tests need to be performed for one new HIV diagnosis, even with physician-directed diagnostic HIV testing [10], our estimated testing rate indicates that EDs in this part of Switzerland are missing undiagnosed HIV infection.

Whilst large studies using rapid HIV tests for non-targeted (opt-out) screening in the ED have shown only modest increases in HIV diagnoses, and often at late stages in the disease [10,11], and whilst the most appropriate model for HIV testing in the ED has yet to be formulated [12,13], a knowledge among ED doctors of the clinical scenarios in which HIV testing is indicated is an important first step. AHI, for example, remains a diagnosis often missed [14,15], whilst being a stage of infection during which the risk of onward HIV transmission is high [16]. The FOPH recommendations clearly set out a list of symptoms and signs of AHI and other HIV-associated pathology and the lack of awareness of these presents a barrier to the diagnostic testing and targeted screening proposed. That said, even if clinicians are aware of these recommendations and apply the testing recommendations perfectly, the targeted approach that the FOPH recommended would fail to identify the majority of undiagnosed HIV infected patients who present to the ED with clinical conditions unrelated to HIV [17,18]. Finally, our observation that senior doctors are aware of the clinical settings in which HIV testing is indicated and yet admit that they would not test in clinical practice deserves further exploration.

Our observations have individual- and public health-level implications. At the individual level, patients who present symptoms and signs which should trigger HIV testing may go untested and ultimately experience the consequences of late diagnosis. At the public health level, missed HIV diagnoses contribute to the risk of onward transmission and indeed to the epidemic itself. For public health professionals, our results suggest a problem with guideline dissemination: of all participants, <3% were aware of the recommendations by way of the original FOPH publication. Finally, if doctors who have read the recommendations do not understand when HIV should be added routinely to other serological tests and do not test the patients they encounter in whom HIV testing is indicated, there are implications regarding not only awareness of recommendations but also agreement and adherence [19].

How can our observations shape clinical practice and health care delivery? Whilst we did not explore the reasons for not testing among our participants (see limitations, below), several barriers to testing in the ED have been cited in other studies. These include internal barriers, such as doctors not perceiving patients to be at risk for HIV infection [20] or not believing that HIV testing is part of their professional remit [21], and external barriers such as cost, lack of time and lack of resources for delivering ‘routine’, as opposed to emergency, care [21] or difficulty in providing follow up care when on-site tests are not available [22]. Our results suggest that potential barriers vary according to the stage of clinical training. For junior doctors who are less aware of testing recommendations than their seniors, universal training in HIV testing in the ED would be beneficial. As ED doctors in Switzerland have a variety of specialist backgrounds, this would allow one aspect of ED training to be unified. For senior doctors, internal barriers could also be addressed in training sessions. Many external barriers to testing could be addressed by simplifying the testing process: by decoupling pre-test counseling from testing for all patients, by verbally informing patients that testing will be performed as part of the diagnostic procedure, and by making available on-site (rapid) testing to optimize continuity of care when issuing the test result. These latter measures are not restricted to Switzerland but are applicable elsewhere: the fine points of national testing recommendations become irrelevant if clinicians perceive barriers – real or imagined – to their application.

This study has limitations. We did not ask participants what training they had received on HIV testing or about their subjective level of competence in this area. For the doctors who identified that HIV testing was indicated but stated a low probability that they would perform a test, we did not ask about perceived barriers to testing. We also did not compare reported testing rates with actual rates, although the low figures obtained from the questionnaire agree with our previous study in one of the five centers [6]. Finally, we did not ask about the methods of HIV testing in each centre, specifically, whether any participants use point-of-care rapid testing and whether this influenced the number of tests requested. As described above, these unexplored areas will form the basis of future studies.

In summary, Swiss ED doctors are not aware of the updated national HIV testing recommendations, do not adhere to its content, and rarely perform HIV tests. Improved dissemination of these recommendations is required if ED doctors are expected to play a significant role in reducing the burden of undiagnosed HIV infection. Furthermore, the identification and tackling of barriers to HIV testing among doctors who recognize when such testing is indicated represents an important parallel step.

## Supporting Information

Table S1 **The principal symptoms and signs indicative of acute HIV infection.**
(DOC)

Table S2 **Clinical indications for HIV screening (a).** Laboratory tests in adults with which HIV screening is indicated (b). Pathologies pathognomonic of AIDS (c).
(DOC)

Table S3 **Indications for HIV screening and counseling as proposed by the doctor.**
(DOC)

Table S4 **Results presented according to emergency department centers participating in the study.**
(DOC)

Text S1 **Translated questionnaire.**
(DOC)
