# Supplementary material for: Awareness of HIV Testing Guidelines Is Low among Swiss Emergency Doctors: A Survey of Five Teaching Hospitals in French-Speaking Switzerland
Source: PLoS One. 2013 Sep 6;8(9):e72812. doi: 10.1371/journal.pone.0072812 (PMC3765151; doi:10.1371/journal.pone.0072812)
Supplement: Table S1 — The principal symptoms and signs indicative of acute HIV infection. (DOC) [file pone.0072812.s001.doc]

### Table S1

The principal symptoms and signs indicative of acute HIV infection

| - Fever |
| --- |
| - Fatigue |
| - Disseminated lymphadenopathy |
| - Erythematous pharyngitis |
| - Rash |
| - Headache |
| - Aseptic meningitis |
| - Reactive lymphocytes |
| Less frequent symptoms: |
| - Myalgia and arthralgia |
| - Acute diarrhea |
| - Nausea and vomiting |
| - Oral or genital ulcers |
| Rare symptoms and signs: |
| - Hepatosplenomegaly |
| - Aseptic meningitis and other neurological manifestations |

### 
