# Supplementary material for: Awareness of HIV Testing Guidelines Is Low among Swiss Emergency Doctors: A Survey of Five Teaching Hospitals in French-Speaking Switzerland
Source: PLoS One. 2013 Sep 6;8(9):e72812. doi: 10.1371/journal.pone.0072812 (PMC3765151; doi:10.1371/journal.pone.0072812)
Supplement: Table S2 — Clinical indications for HIV screening (a). Laboratory tests in adults with which HIV screening is indicated (b). Pathologies pathognomonic of AIDS (c). (DOC) [file pone.0072812.s002.doc]

### Table S2a

Clinical indications for HIV screening

| Mononucleosis-like illness (cf Table S1) |
| --- |
| Sexually transmitted infection (syphilis, gonorrhea, Chlamydia, lymphogranuloma venerium, genital herpes, hepatitis A, B and C) |
| Neurological pathology (dementia, meningitis, encephalitis, facial palsy, polyneuropathy) |
| Hodgkin’s lymphoma |
| Mucucutaneous lesions caused by *Candida albicans,* herpes zoster in individuals <40 years old, seborrheic dermatitis, oral hairy leucoplakia, unexplained exanthema, generalized peripheral lymphadenopathy, anal carcinoma, cervical dysplasia |
| Cachexia syndrome |
| Pregnancy |
| Organ, blood or sperm donation |
| Pathologies pathognomonic of AIDS (cf Table S2c) |

### Table S2b

Laboratory tests in adults with which HIV screening is indicated

| Epstein-Barr virus serology |
| --- |
| Cytomegalovirus serology |
| Toxoplasmosis serology, depending on clinical indication |
| Syphilis screening |
| Hepatitis B or C virus screening |
| Serology for measles, rubella, etc (unexplained exanthem with fever in an adult) |

### Table S2c

Pathologies pathognomonic of AIDS

| Tuberculosis |
| --- |
| Esophageal candidiasis |
| *Pneumocystis jiroveci* pneumonia (formerly *P. carinii*) |
| Recurrent bacterial pneumonia |
| Chronic herpes simplex lesions |
| Kaposi’s sarcoma |
| Cervical carcinoma |
| Chronic diarrhea caused by cryptosporidium or *Isospora belli* |
| Progressive multifocal leucoencephalopathy |
| CNS toxoplasmosis |
| Primary CNS lymphoma |
| Non-Hodgkin lymphoma |
| Cryptococcal meningitis |
| CMV retinitis, esophagitis or colitis |
| Disseminated atypical mycobacterial disease |
| Extrapulmonary histoplasmosis or coccidioidomycosis |

### 
