# Supplementary material for: Awareness of HIV Testing Guidelines Is Low among Swiss Emergency Doctors: A Survey of Five Teaching Hospitals in French-Speaking Switzerland
Source: PLoS One. 2013 Sep 6;8(9):e72812. doi: 10.1371/journal.pone.0072812 (PMC3765151; doi:10.1371/journal.pone.0072812)
Supplement: Table S3 — Indications for HIV screening and counseling as proposed by the doctor. (DOC) [file pone.0072812.s003.doc]

### Table S3

Indications for HIV screening and counseling as proposed by the doctor

| The patient is from a high risk group: men who have sex with men, injecting drug users, individuals from countries with high HIV seroprevalence (sub-Saharan Africa) |
| --- |
| The patient describes high risk sexual behavior with a person known to be HIV positive or in a setting where HIV seroprevalence is high |
| A person who is sexually active and wishes to be tested |
| A person returning from travels who wishes to be tested |
