# Supplementary material for: Awareness of HIV Testing Guidelines Is Low among Swiss Emergency Doctors: A Survey of Five Teaching Hospitals in French-Speaking Switzerland
Source: PLoS One. 2013 Sep 6;8(9):e72812. doi: 10.1371/journal.pone.0072812 (PMC3765151; doi:10.1371/journal.pone.0072812)
Supplement: Table S4 — Results presented according to emergency department centers participating in the study. (DOC) [file pone.0072812.s004.doc]

### Table S4.

Results presented according to emergency department centers participating in the study: patient visits per annum, number of doctors, study participants, questionnaire scores (percentage of correct responses), number of HIV tests requested by participants, and awareness of national HIV testing recommendations. Abbreviations: IQR, interquartile range; SD, standard deviation

| Characteristic | Center 1 | Center 2 | Center 3 | Center 4 | Center 5 | All centers |
| --- | --- | --- | --- | --- | --- | --- |
| University hospital | Yes | Yes | No | No | No |  |
| Patient visits per annum, n | 36,704 | 59,873 | 27,953 | 21,304 | 29,544 | 175,378 |
| Doctors employed in ED, n | 60 | 44 | 22 | 17 | 21 | 164 |
| Study Participants, n (%) | 60 (100) | 40 (91) | 22 (100) | 13 (77) | 9 (43) | 144 (88) |
| Percentage of total (n=144) | 42 | 28 | 15 | 9.0 | 6.3 | 100 |
| Participant breakdown by grade, n (%): |  |  |  |  |  |  |
| Resident | 36 (60) | 21 (53) | 14 (64) | 7 (54) | 5 (56) | 83 (58) |
| Chief Resident | 13 (22) | 14 (35) | 3 (14) | 4 (31) | 2 (22) | 36 (25) |
| Attending | 11 (18) | 5 (13) | 5 (23) | 2 (15) | 2 (22) | 25 (17) |
| Postgraduate experience, years, median (IQR) | 7.3 (4;12) | 6.5 (3;11) | 4.3 (2;12) | 4.6 (3;11) | 2.5 (2;5) | 6.5 (3;12) |
| Questionnaire score (% correct responses): |  |  |  |  |  |  |
| Section 1 : scenarios, mean (SD) | 66 (15) | 65 (13) | 68 (14) | 68 (17) | 62 (15) | 66 (14) |
| Section 2 : consent, mean (SD) | 46 (16) | 37 (16) | 36 (16) | 30 (15) | 37 (10) | 40 (16) |
| Section 2 : serology, mean (SD) | 71 (17) | 75 (18) | 58 (17) | 64 (22) | 67 (19) | 69 (18) |
| Total (Sections 1+2), mean (SD) | 62 (12) | 60 (10) | 57 (9.3) | 56 (14) | 56 (9.5) | 59 (11) |
| Number of tests in past month, median (IQR) | 0.5 (0;2) | 1 (0;1) | 0 (0;2) | 1 (0;2) | 1 (0;2) | 1 (0;2) |
| Awareness of recommendations, n (%) | 16/60 (27) | 6/40 (15) | 4/22 (18) | 0/13 (0) | 0/9 (0) | 26/144 (18) |
