# Supplementary material for: Awareness of HIV Testing Guidelines Is Low among Swiss Emergency Doctors: A Survey of Five Teaching Hospitals in French-Speaking Switzerland
Source: PLoS One. 2013 Sep 6;8(9):e72812. doi: 10.1371/journal.pone.0072812 (PMC3765151; doi:10.1371/journal.pone.0072812)
Supplement: Text S1 — Translated questionnaire. (DOC) [file pone.0072812.s005.doc]

**Text S1.** Translated questionnaire

**KNOWLEDGE & APPLICATION OF HIV TESTING RECOMMENDATIONS IN THE EMERGENCY DEPARTMENT (ED)**

| Personal data:  Year of birth: ……….  Gender: □ Female □ Male  Title: □ Resident  □ Chief Resident  □ Attending  Date of qualification (month & year): ………………....  Percentage of time in ED: ……………………  Holding FMH (post-graduate specialist qualification): ………………………………………………………  Training for FMH: ……………………………………………………… |
| --- |

**Section 1:**

In this section, please indicate whether, according to the HIV testing recommendations published in 2010 by the Federal Office of Public Health (FOPH), you should perform an HIV test in the clinical situations below. Please then indicate for each situation the probability with which you would perform an HIV test in clinical practice.

1. A 35 year old patient presents with peripheral facial palsy.

- The guidelines recommend HIV testing in this situation:

□ yes □ no

- In practice, what is the probability you would request an HIV test in this setting:

□ 0-25 % □ 26-50 % □ 51-75 % □ 76-100 %

1. A 28 year old woman presents to the ED with a 3-day history of a red, sore throat, fever of 38.2°C and swollen submandibular lymph nodes, but without associated cough or sputum production.

- The guidelines recommend HIV testing in this situation:

□ yes □ no

- In practice, what is the probability you would request an HIV test in this setting:

□ 0-25 % □ 26-50 % □ 51-75 % □ 76-100 %

1. A 66 year old man presents to the ED with a 2-day history of fever of 39°C and headaches. Clinical examination reveals nuchal rigidity and CSF Gram stain shows Gram negative diplococci.

- The guidelines recommend HIV testing in this situation:

□ yes □ no

- In practice, what is the probability you would request an HIV test in this setting:

□ 0-25 % □ 26-50 % □ 51-75 % □ 76-100 %

1. A 30 year old woman presents with weight loss of 5 Kg over the past 3 months, with diarrhoea and intermittent fever.

- The guidelines recommend HIV testing in this situation:

□ yes □ no

- In practice, what is the probability you would request an HIV test in this setting:

□ 0-25 % □ 26-50 % □ 51-75 % □ 76-100 %

1. An 80 year old woman with a urinary catheter is brought to the ED by her daughter with fever and confusion. You make a diagnosis of urosepsis.

- The guidelines recommend HIV testing in this situation:

□ yes □ no

- In practice, what is the probability you would request an HIV test in this setting:

□ 0-25 % □ 26-50 % □ 51-75 % □ 76-100 %

1. A 53 year old cleaner presents to the ED with a several-month history of general fatigue.

- The guidelines recommend HIV testing in this situation:

□ yes □ no

- In practice, what is the probability you would request an HIV test in this setting:

□ 0-25 % □ 26-50 % □ 51-75 % □ 76-100 %

1. A 34 year old man presents to the ED following trauma to the wrist. An x-ray shows a non-displaced fracture of the distal radius.

- The guidelines recommend HIV testing in this situation:

□ yes □ no

- In practice, what is the probability you would request an HIV test in this setting:

□ 0-25 % □ 26-50 % □ 51-75 % □ 76-100 %

1. A 47 year old woman presents to the ED with neck stiffness and is sub-febrile at 37.8°C. CSF examination reveals nothing on Gram stain and CSF glucose is normal, but CSF protein is raised and white cell count is 50 cells/mm^3^, predominantly lymphocytes.

- The guidelines recommend HIV testing in this situation:

□ yes □ no

- In practice, what is the probability you would request an HIV test in this setting:

□ 0-25 % □ 26-50 % □ 51-75 % □ 76-100 %

1. A 28 year old man complains of dysuria and erythema of the urethral meatus with no history of at-risk sexual contact.

- The guidelines recommend HIV testing in this situation:

□ yes □ no

- In practice, what is the probability you would request an HIV test in this setting:

□ 0-25 % □ 26-50 % □ 51-75 % □ 76-100 %

1. A 45 year old patient, vaccinated against measles, presents to the ED with a rash and is subfebrile at 37.6°C.

- The guidelines recommend HIV testing in this situation:

□ yes □ no

- In practice, what is the probability you would request an HIV test in this setting:

□ 0-25 % □ 26-50 % □ 51-75 % □ 76-100 %

1. A 35 year old patient presents with painful vesicles over the trunk in the distribution of dermatome T10.

- The guidelines recommend HIV testing in this situation:

□ yes □ no

- In practice, what is the probability you would request an HIV test in this setting:

□ 0-25 % □ 26-50 % □ 51-75 % □ 76-100 %

1. A young patient of 19 presents with nausea and vomiting. A pregnancy test is positive.

- The guidelines recommend HIV testing in this situation:

□ yes □ no

- In practice, what is the probability you would request an HIV test in this setting:

□ 0-25 % □ 26-50 % □ 51-75 % □ 76-100 %

**Section 2:**

Please respond to the questions below according to the 2010 HIV testing recommendations.

1. What would you do if faced with a 39 year old patient presenting to the ED with typical clinical features of acute HIV infection (seroconversion) with a history of unprotected intercourse with his wife: (**1 answer allowed**)
   - You inform him about how contagious this infection is at this stage of the disease and you inform him that you are going to perform an HIV test.
   - As the patient is not in an at-risk group for HIV, you do not recommend the test but refer him to his general practitioner for this problem.
   - You inform him about the risks of transmission at this stage of the disease and offer him an HIV test after providing pre-test information, the test itself being carried out only with the patient’s agreement.
2. In the ED, you suspect that HIV infection is the cause of your patient’s presenting pathology: **(1 answer allowed)**
   - You request the explicit agreement of the patient before conducting an HIV test.
   - You must inform the patient that you are going to carry out an HIV test.
   - It is recommended that the patient is informed that an HIV test is going to be conducted.
   - Pre-test counseling applies in this situation.

In which situation(s) is pre-test counseling indicated according to the HIV testing recommendations?

1. If the patient expresses the wish to take the HIV test.

□ yes □ no

1. If the patient tells you he is homosexual.

□ yes □ no

1. If you are faced with the clinical features of acute HIV infection.

□ yes □ no

1. If you are faced with a patient suffering from Kaposi’s sarcoma.

□ yes □ no

1. If the patient is from Sub-Saharan Africa

□ yes □ no

**Section 2 (continued):**

With which serological tests should you automatically request an HIV test? (**several answers possible**)

1. Screening for hepatitis B

□ yes □ no

1. Screening for hepatitis A

□ yes □ no

1. Lyme for unexplained AV block

□ yes □ no

1. EBV

□ yes □ no

1. CMV

□ yes □ no

1. Measles for an unexplained rash

□ yes □ no

**Section 3:**

Please respond to the questions below.

Please state how many HIV tests have you requested during the last 4 weeks of professional activity in the ED:

……………………………………………………………………………

Are you aware of the latest recommendations on HIV testing published in March 2010 in the FOPH bulletin?

□ yes □ no

If yes, by which means:

- - Clinical seminar
  - On line search (Pubmed, or other)
  - The FOPH Bulletin
  - The Swiss Medical Forum article of 2010: *HIV infection – early testing limits damage for the individual and for society*
  - Other: ………………………………………...
